# Identification of microRNA-Related Target Genes for the Development of Otic Organoids

**DOI:** 10.3390/ijms262110627

**Published:** 2025-10-31

**Authors:** Sehee Lee, Sungjin Park, Euyhyun Park, Gi Jung Im, Jiwon Chang

**Affiliations:** 1 Department of Otorhinolaryngology-Head & Neck Surgery, Korea University Anam Hospital, 73 Goryeodae-ro, Seongbuk-gu, Seoul 02841, Republic of Korea; lshe5@korea.ac.kr (S.L.); randoll@korea.ac.kr (E.P.); logopas@korea.ac.kr (G.J.I.); 2 Institute of Human Behavior & Genetics, College of Medicine, Korea University, 73 Goryeodae-ro, Seongbuk-gu, Seoul 02841, Republic of Korea; spark42@korea.ac.kr; 3 Precision Medicine Center, Korea University Anam Hospital, 73 Goryeodae-ro, Seongbuk-gu, Seoul 02841, Republic of Korea

**Keywords:** inner ear organoid, differentiation, development, regeneration, extracellular vesicles, miRNAs

## Abstract

Mammalian hearing loss is typically permanent due to the inability to replace damaged cochlear hair cells. However, the neonatal mice inner ear demonstrates regenerative capacity, with cochlear floor cells proliferating and differentiating into organoids containing new hair cells and supporting cells, yet the governing molecular mechanisms remain poorly understood. Here, we isolated extracellular vesicles (EVs) from inner ear organoids at proliferation and differentiation stages, characterized their EV miRNA profiles through sequencing, and validated findings using public transcriptomic datasets to elucidate miRNA-mediated regulatory mechanisms during inner ear development. Inner ear organoids were successfully developed from cochlear duct cells, expressing otic progenitor marker SOX2 and hair cell marker Myo7A and demonstrating functional mechano-transduction activity through FM1-43 uptake. Small RNA sequencing identified 35 differentially expressed EV miRNAs between developmental stages. Integrated analysis with public transcriptome datasets revealed 18 genes with significant differential expression, leading to identification of three key regulatory genes—*Trp53*, *Ezh2*, and *Zbtb4*—that exhibited dynamic spatiotemporal expression during inner ear maturation. Pathway analysis demonstrated that these genes are associated with DNA Repair, P53, and Wnt/β-Catenin signaling with remarkable cell-type specificity. Our results demonstrate that EV miRNAs are temporally regulated during organoid development, with predominant downregulation during differentiation. These findings provide crucial insights into developmental mechanisms that could optimize organoid-based models and guide EV miRNA-based therapeutic strategies for hearing restoration.

## 1. Introduction

The irreversibility of sensory hair cells in the mammalian inner ear remains a principal cause of permanent hearing loss as these cells exhibit minimal regenerative capacity in postnatal and adult stages. The mammalian cochlear cells lack the regenerative ability to replace lost hair cells in contrast to the avian cochlea, where the non-sensory cells enter the cell cycle in response to the damage and regenerate new hair cells [1,2]. However, recent studies have shown that neonatal non-sensory epithelial cells of the mouse cochlea, limited to the first two neonatal weeks, exhibit a potential for generation of inner ear hair cells in vivo and in vitro and grow organoids in a culture medium supplemented with a combination of epigenetic modifiers and growth factors [3,4,5,6,7]. Organoid cells eventually differentiate into new sensory hair cells and associated non-sensory cells. Moreover, recent studies have reported the successful generation of inner ear organoids using embryonic pluripotent stem cells and induced pluripotent stem cells (iPSCs) by recapitulating the in vivo development process [8,9,10]. The development of the inner ear organoid model has provided a powerful in vitro platform to restate key aspects of inner ear development and regeneration.

Extracellular vesicles (EVs) are phospholipid-membrane-enclosed particles released from cells into the extracellular space and play a role in intracellular communication by transferring their components, including proteins, metabolites, and nucleic acids, such as miRNAs [11,12]. miRNAs are one of the most abundant RNA species in EVs. They are noncoding RNAs that regulate gene expression and are key regulators of many biological processes during embryogenesis, development, homeostasis, and regeneration. They regulate self-renewal, pluripotency, proliferation, and differentiation of various stem cells and progenitor cells by modulating critical transcription factor expression [13,14,15]. In the field of inner ear research, miRNA expressions have been identified in mouse and human inner ear sensory epithelial cells during embryonic and postnatal development [16,17,18]. Conditional knockout experiments targeting specific miRNAs resulted in developmental defects in the inner ear, emphasizing the crucial roles of miRNAs in the development. In our previous study, we have focused on characterizing the miRNAs in EVs released by inner ear organoids, hypothesizing their proliferating role during organoid growth [7]. While organoid-derived miRNA profiling reflects intracellular regulatory dynamics, we focused on extracellular-vesicle-derived miRNAs to capture signals mediating intercellular communication during organoid growth, particularly those influencing proliferation, differentiation, and development.

In this study, we established otic organoids from neonatal mouse cochlear progenitor cells and harvested EVs at both the proliferation and differentiation-stage organoids. We based our investigation on the hypothesis that miRNAs contained in either the proliferation or differentiation-stage organoid-derived EVs are diverse and could work efficiently according to developmental stages. We also focused on EV miRNAs differentially expressed in differentiation-stage organoids to elucidate the key aspects of sensory hair cell development and regeneration. By employing high-throughput miRNA sequencing and integrative bioinformatics analysis, we sought to identify differentially expressed EV miRNAs and their putative target genes that may regulate key developmental transitions in otic organoid formation. Furthermore, by leveraging both our own experimental data and public transcriptomic datasets, we aimed to construct a comprehensive regulatory network governing inner ear organoid development. Elucidation of these EV miRNA-mediated pathways may not only enhance our understanding of the intrinsic regenerative mechanisms of the mammalian inner ear but also facilitate the optimization of organoid-based models for translational research and therapeutic development.

## 2. Results

### 2.1. Generation of Inner Ear Organoids from Cochlear Duct Cells

**Figure 1:**
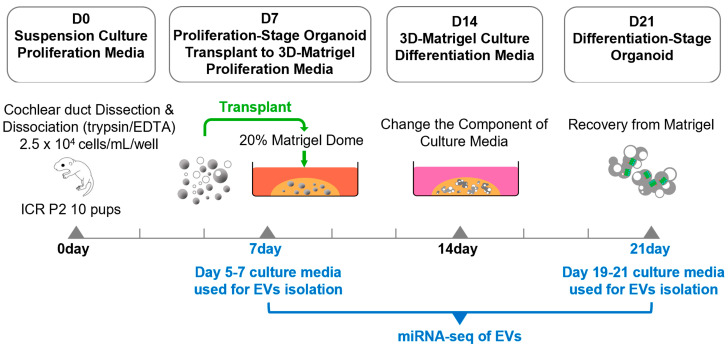
Workflow for EV generation from proliferation and differentiation-stage organoids. Organoids were grown from dissociated cochlear ducts. Dissociated cells were seeded at densities of 2.5 × 10^4^ cells/mL in 12-well suspension culture dishes and maintained in proliferation medium at 37 °C and 5% CO_2_. Half of the medium was replaced every other day, and the cells were cultured for 7 days. On day 7, the proliferation-stage organoids were transplanted into a 20% Matrigel dome and were cultured for 7 more days under the same medium conditions, and then they were changed to a differentiation medium and cultured for another 7 days. EVs were isolated from the 5th–7th-day and 19th–21st-day culture media. EVs isolated from both groups were subjected to miRNA sequencing analysis.

**Figure 2:**
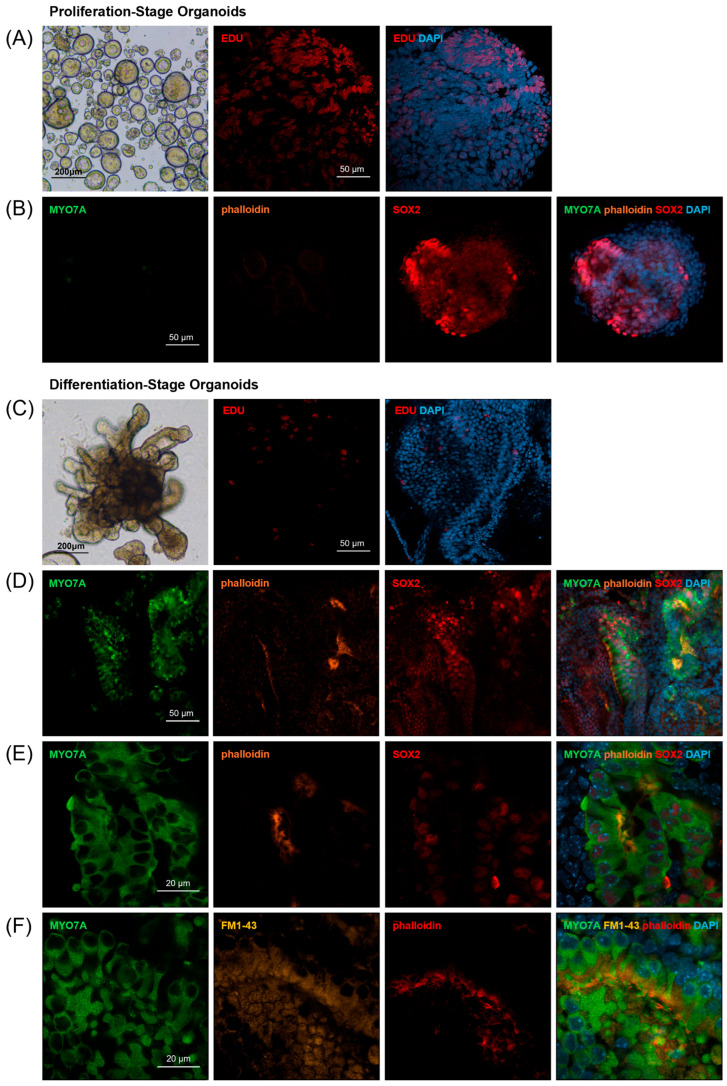
Organoid formation and characterization. (**A**,**B**) Proliferation-stage organoids. (**A**) Day 7 organoids were round and solid morphologies (scale bar 200 µm). Nuclear EdU incorporation was observed in proliferation-stage organoids at day 7 (scale bar 50 µm). (**B**) The expression of SOX2 was observed, but Myo7A or phalloidin-expressing cells were not observed in the organoids on day 7 (scale bar 50 µm). (**C**–**F**) Differentiation-stage organoids. (**C**) Day 21 organoids formed multiple branches (scale bar 200 µm) and were decreased in EdU incorporation (scale bar 50 µm). (**D**,**E**) The expression of Myo7A, phalloidin, and SOX2 was observed in differentiation-stage organoids on day 21 (scale bar 50 µm, 20 µm). (**F**) Sensory hair cells with intact stereociliary bundles (phalloidin) and mechano-transduction channel activity (FM1-43 uptake) were observed on day 21 (scale bar 20 µm).

Dissociated cochlear duct cells from postnatal day two mice proliferate and grow into floating spherical organoids when cultured in proliferation media enriched with growth factors and small molecules for seven days [4,19,20]. After switching the culture conditions to adhesive 20%-Matrigel and differentiation media, a differentiation process starts, leading to the development of sensory epithelium-like structures featuring hair cell- and supporting cell-marker-expressing cells (Figure 1) [6,20]. Previously, we have determined the optimal culture condition for generating proliferating-stage organoids [21]; therefore, after seeding cochlear duct cells at cell densities of 2.5 × 10^4^ cells/mL in suspension culture, we observed solid spherical organoids (Figure 2A). To identify that a significant portion of organoid cells are proliferating at days 5–6 in vitro, we added EdU (5-ethynyl 2′-deoxyuridine) to the culture media and assessed nuclear EdU incorporation on day seven with a population of labeled organoid cells, further confirming that cell proliferation was driving organoid growth (Figure 2A). Additionally, we detected the expression of the otic progenitor marker SOX2, but the hair cell markers Myo7A and phalloidin were not detected on day seven, indicating that the differentiation of hair-cell-marker-expressing cells has not developed yet (Figure 2B).

Switching the culture conditions initiates a differentiation process over the subsequent 14 days, resulting in sensory epithelium-like structures featuring hair cell- and supporting cell-marker-expressing cells. Spherical organoids grew in their size, elongated, and formed multiple branches after 14 days (Figure 2C). Immunohistochemistry performed on organoids on culture day 21 demonstrated decreased nuclear EdU incorporation, suggesting that cell proliferation was reduced in this differentiation stage (Figure 2C). Also, immunohistochemistry performed on culture day 21 not only exhibited the expression of the otic progenitor marker SOX2 but also the hair cell markers Myo7A and phalloidin (Figure 2D,E). Additionally, we detected a large number of hair cells with intact stereociliary bundles and mechano-transduction channel activity as indicated by FM1-43 uptake (Figure 2F) [22].

### 2.2. Isolation of EVs from Proliferation and Differentiation-Stage Organoids

**Figure 3:**
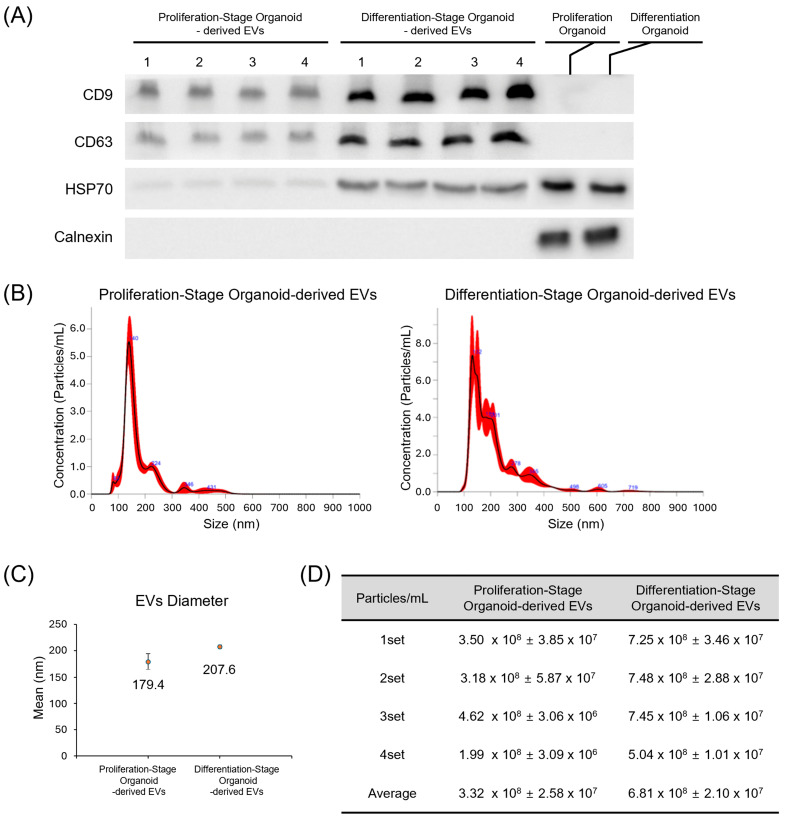
Characterization of EVs. (**A**) Presence of EV markers CD9, CD63, and Hsp70 and the marker for cell-derived endoplasmic reticulum calnexin was assessed in both proliferation and differentiation-stage organoids. Lysates from organoids were individually used to detect calnexin as positive controls, which indicates the presence of cellular material. (**B**) Size distribution of proliferation and differentiation-stage organoid-derived EVs. (**C**) The average diameters of proliferation and differentiation-stage organoids. (**D**) The numbers of EVs contained in one mL volume of organoid-culturing media (N = 4).

EVs were isolated from organoid culture media between culture days 5–7 and day 19–21 using an aqueous two-phase system (exosomeplus.com) (Figure 1). We confirmed the expression of exosome markers CD9, CD63, and Hsp70 in EV preparations from both proliferation and differentiation-stage organoids, while the cell-specific marker calnexin was absent (Figure 3A). This control experiment confirmed that we isolated EVs and not cellular material. Nanoparticle tracking analysis (NTA) revealed that the average size of EVs derived from proliferation and differentiation-stage organoids was 179.4 ± 15.2 nm and 207.6 ± 3.4 nm, respectively (Figure 3B,C). The average concentrations of these EVs were measured at 3.32 × 10^8^ particles/mL for proliferation-stage organoids and 6.81 × 10^8^ particles/mL for differentiation-stage organoids (Figure 3D).

### 2.3. Differential Expression of EV miRNAs and Target Gene Networks in Inner Ear Organoid Development

**Figure 4:**
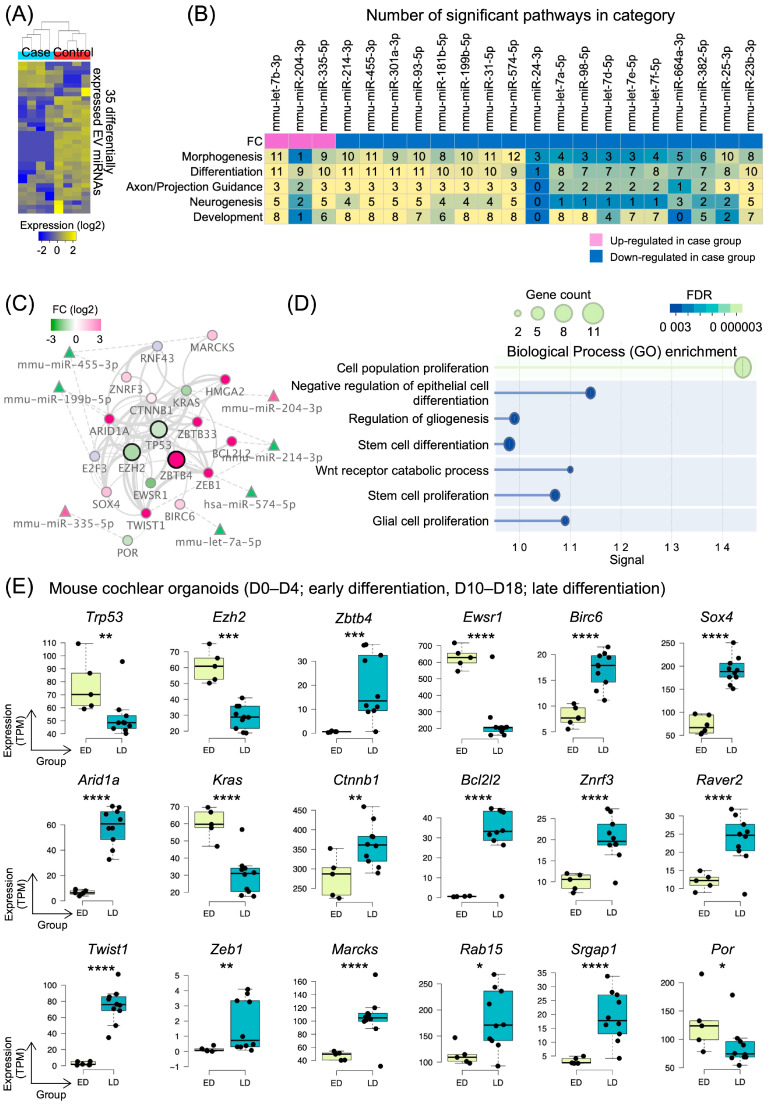
EV miRNA expression and target gene networks in inner ear organoid differentiation. (**A**) Heatmap showing 35 differentially expressed miRNAs in extracellular vesicles isolated from differentiation-stage organoids (case) compared to proliferation-stage organoids (control). (**B**) Heatmap displaying the number of significant pathways in five major categories (morphogenesis, differentiation, axon/projection guidance, neurogenesis, and development) regulated by 21 differentially expressed EV miRNAs. Pink and blue colors indicate up and downregulation in the case group, respectively. (**C**) Protein–protein interaction network of target genes regulated by differentially expressed EV miRNAs. The network is centered around TP53 (highest connectivity degree ~480) and shows key interaction partners including EZH2, ZBTB4, and other regulatory proteins, with node colors indicating log2 fold-change. (**D**) Dot plot showing enriched biological processes (GO terms) in the protein interaction network, including cell population proliferation, stem cell differentiation, and Wnt receptor catabolic process. Dot size represents gene count and color intensity indicates FDR significance. (**E**) Box plots showing expression levels of 18 differentially expressed genes between early differentiation (ED) and late differentiation (LD) stages from bulk gene expression datasets, including downregulated genes (*Trp53* and *Ezh2*) and upregulated genes (*Zbtb4*, *Sox4*, and *Ctnnb1*). Significance levels are indicated by asterisks (* *p* < 0.05, ** *p* < 0.01, *** *p* < 0.001, and **** *p* < 0.0001).

To investigate the role of EV miRNAs in inner ear organoid development, we performed small RNA sequencing of EV miRNAs from organoids at two distinct developmental stages: proliferation-stage organoids (control) and differentiation-stage organoids (case). Small RNA sequencing of these EVs revealed 35 differentially expressed miRNAs between the two groups (Figure 4A). Among these, 6 miRNAs were significantly upregulated in the differentiation stage, while 29 miRNAs showed significant downregulation, suggesting a predominant reduction in specific miRNA secretion during the transition from proliferation to differentiation. The complete list of differentially expressed miRNAs is provided in Supplementary Figure S1. And the orthologies of differentially expressed mouse miRNAs are listed in Supplementary Table S1.

To understand the biological implications of these differentially expressed EV miRNAs, we performed pathway analysis using an established miRNA database. Of the 35 differentially expressed miRNAs, 21 were significantly associated with 92 gene ontology biological processes (Supplementary Figure S2). These biological processes were categorized into five major groups relevant to cochlear development: morphogenesis, differentiation, axon/projection guidance, neurogenesis, and development (Figure 4B). The heatmap analysis revealed that individual miRNAs regulated distinct numbers of pathways across these categories, with certain miRNAs such as mmu-let-7b-3p, mmu-miR-455-3p, and mmu-miR-574-5p affecting a larger number of pathways than others.

We subsequently constructed a protein–protein interaction network comprising 8468 target mRNAs of these 21 EV miRNAs. The largest submodule contained 5900 protein nodes (Supplementary Figure S4A). From this comprehensive network, we analyzed the distribution of proteins by functional family (Supplementary Figure S4B), which revealed that enzymes constituted the largest category (>1400 proteins), followed by transcription factors, kinases, and transporters. The “Other” category comprised approximately more than 3000 proteins with diverse functions. To identify the key regulatory nodes within this network, we performed degree distribution analysis across different protein families (Supplementary Figure S4C). This analysis highlighted human protein TP53 as the most highly connected protein node with a connectivity degree of approximately 480, followed by other critical regulator human proteins, including MYC, HDAC1, STAT3, EP300, JUN, HDAC2, EZH2, CREBBP, and NFKB1. From this comprehensive network, we extracted a subnetwork of proteins involved in cell differentiation, with TP53 identified as the most highly connected node. This TP53-centered network included key human regulatory proteins involved in cochlear development, such as EZH2 and ZBTB4, and several other interacting partners (Figure 4C).

Gene Ontology enrichment analysis of this subnetwork revealed significant associations with biological processes critical for cochlear development, including cell population proliferation, negative regulation of epithelial cell differentiation, regulation of gliogenesis, stem cell differentiation, Wnt receptor catabolic process, stem cell proliferation, and glial cell proliferation (Figure 4D). These processes are known to be essential for proper inner ear development and sensory epithelium formation.

Finally, we validated the expression patterns of the mouse orthologs of these key human network genes using publicly available bulk gene expression datasets from cochlear tissues (Figure 4E). This dataset was constructed through trajectory analysis of two mouse organoid gene expression datasets, incorporating only samples connected in pseudo-time order based on differentiation days (Supplementary Figure S3). We identified 18 mouse genes with significant differential expression between early differentiation (ED) and late differentiation (LD) stages. Among these, 5 mouse genes including *Trp53*, *Ezh2*, and *Ewsr1* were significantly downregulated in the late differentiation stage, while 13 mouse genes including *Zbtb4, Sox4*, and *Ctnnb1* were significantly upregulated.

### 2.4. Cell-Type-Specific Expression Dynamics of Key Regulatory Genes During Inner Ear Development

**Figure 5:**
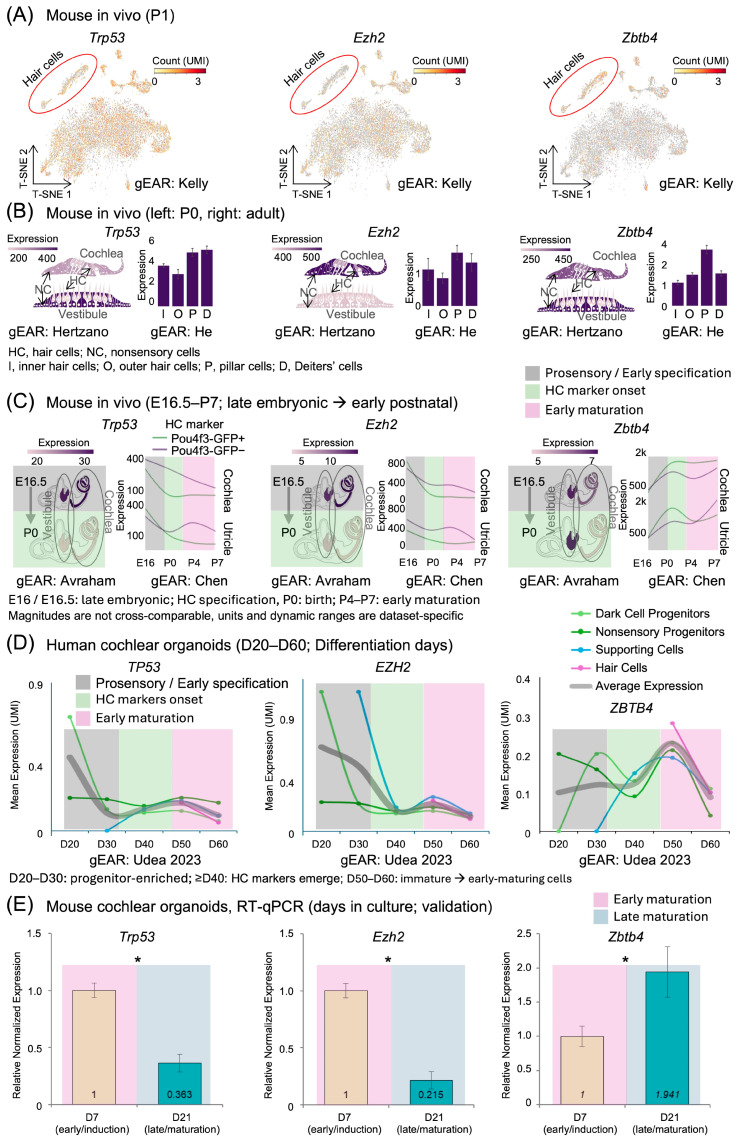
Spatiotemporal expression patterns of *Trp53*, *Ezh2*, and *Zbtb4* during inner ear development. (**A**) t-SNE plots showing expression of *Trp53*, *Ezh2*, and *Zbtb4* in mouse cochlear cells. Red circles indicate hair cells. Color intensity represents UMI count. Data visualized from the Kelly dataset in the gEAR portal. (**B**) Cell-type-specific expression of *Trp53*, *Ezh2*, and *Zbtb4* in cochlear and vestibular tissues (Hertzano dataset, left panels) and in specific cochlear cell types (He dataset, right panels). Left panels show schematic illustrations of each tissue with expression levels indicated by color intensity. Right panels show bar graphs of expression levels in inner hair cells (I), outer hair cells (O), pillar cells (P), and Deiters’ cells (D). HC, hair cells; NC, nonsensory cells. (**C**) Developmental expression profiles of *Trp53*, *Ezh2*, and *Zbtb4* in mouse inner ear. Left panels show schematic illustrations of inner ear at embryonic day 16.5 (E16.5) and postnatal day 0 (P0) from the Avraham dataset. Right panels show expression trends in cochlea and utricle from embryonic day 16 (E16) to postnatal day 7 (P7) in Pou4f3-GFP positive (hair cells) and negative (non-hair cells) populations from the Chen dataset. (**D**) Expression levels of *TP53*, *EZH2*, and *ZBTB4* in human cochlear organoids from day 20 to day 60 of differentiation from the Udea 2023 dataset. Different colors represent distinct cell types: dark cell progenitors, nonsensory progenitors, supporting cells, and hair cells. (**E**) RT-PCR quantitative analysis of relative normalized expression in our cultured inner ear organoids comparing day 7 (D7) and day 21 (D21), showing significant downregulation of *Trp53* (0.363-fold) and *Ezh2* (0.215-fold) and upregulation of *Zbtb4* (1.941-fold) (* *p* < 0.05). Time is shown in native units for each dataset (E16–P0; E16.5–P7; D20–D60; D7/21), and expression magnitudes are not cross-comparable across panels. Phase overlaid background indicate approximate developmental equivalence used for qualitative comparisons; see Supplementary Table S2 for the time-to-phase mapping. All datasets (A–D) were accessed and visualized through the Gene Expression Analysis Resource (gEAR; https://umgear.org/NIHL, accessed on 20 February 2025) portal as described in Table 1.

Based on our network analysis, we further investigated the cell-type-specific expression patterns of three key human regulatory genes—*TP53*, *EZH2*, and *ZBTB4*—and their respective mouse orthologs—*Trp53*, *Ezh2*, and *Zbtb4*—across developmental stages in both mouse cochlear tissues and human cochlear organoids. T-SNE visualization of single-cell RNA sequencing data from mouse cochlear cells revealed distinct expression patterns for these mouse genes (Figure 5A). In the mouse tissues, hair cells, highlighted by red circles, showed lower expression of *Trp53* and *Ezh2* compared to other cell types, while *Zbtb4* exhibited higher expression in hair cells compared to other cell types.

A detailed analysis of cell-type-specific expression in mouse tissues revealed that the mouse genes *Trp53* and *Ezh2* were expressed at higher levels in nonsensory cells (NCs) compared to hair cells (HCs) in the cochlea (Figure 5B). The mouse gene *Trp53* showed higher expression in the vestibule compared to the cochlea, while *Ezh2* exhibited higher expression in the cochlear compared to the vestibule. Both mouse genes displayed elevated expression in pillar cells (P) and Deiters’ cells (D), compared to inner (I) and outer (O) hair cells. In contrast, the mouse gene *Zbtb4* showed comparable expression between cochlear and vestibule, with peak expression in pillar cells.

We compared datasets from mouse cochlear tissues (E16.5–P0 and E16–P7), human cochlear organoid (D20–D60), and RT-qPCR of mouse cochlear organoid (D7 and D21) along a common biological trajectory rather than by absolute time. To aid interpretation without temporal rescaling, Figure 5 annotates each panel with developmental phase labels—prosensory/early specification, onset of HC features, and early and late maturation—and our cross-dataset statements refer to state equivalence across these phases.

The directional trends were consistent across datasets. Expression of the human gene *TP53* and its mouse ortholog *Trp53*, as well as the human gene *EZH2* and its mouse ortholog *Ezh2*, decreased from early to later phases. Conversely, expression of the human gene *ZBTB4* and its mouse ortholog *Zbtb4* increased in the later stages. Temporal analysis of gene expression in mouse inner ear tissues revealed dynamic regulation patterns during maturation (Figure 5C). In these mouse tissues, the expression levels of the mouse genes *Trp53* and *Ezh2* decreased markedly from embryonic day 16.5 (E16.5) to postnatal day 0 (P0) in both vestibular and cochlear regions. This downregulation pattern of the mouse genes persisted throughout later developmental stages, from embryonic day 16 (E16) to postnatal day 7 (P7), in both hair cells (Pou4f3-GFP positive) and non-hair cells (Pou4f3-GFP negative) of the cochlea and utricle. The mouse gene *Zbtb4* showed a more complex pattern, with initial low expression that increased during later developmental stages, particularly in cochlear tissues.

**Table 1:** Summary of public datasets used in this study.

	Database: Accession ID	Number of Samples	Description
Bulk RNA-Seq Datasets for Differential Expression Analysis	GEO: GSE132635 [23]	n = 18	This dataset comprises RNA-sequencing data from mouse cochlear organoids at various stages of differentiation. Lgr5-positive cochlear progenitors were harvested from newborn mice, cultured in Matrigel, and sampled at four timepoints during differentiation (D0, D2, D4, and D10). In parallel, native cochlear cells were isolated from transgenic mice expressing fluorescent markers for hair cells (Atoh1-GFP), supporting cells (Lgr5-GFP), and progenitor cells (Sox2-GFP). All samples were sequenced on an Illumina NextSeq 500 platform with single-end 75bp reads. The dataset includes 18 samples in total, with biological duplicates for each condition, allowing for comparison between native cell populations and organoid samples across the differentiation timeline.
	GEO: GSE243581 [24]	n = 8	This dataset contains RNA-sequencing data from mouse cochlear organoids specifically focused on metabolic changes during differentiation. Cochlear sensory epithelia were isolated from P3 mice and cultured as explants with various small molecule treatments. The study compared proliferating organoids (day 10) with differentiated organoids (day 18) to identify changes in oxidoreductase expression and metabolic pathways. All samples were sequenced on the Illumina NovaSeq 6000 platform. The dataset includes 8 samples in total: 4 biological replicates of proliferation-stage organoids (day 10) and 4 biological replicates of differentiation-stage organoids (day 18). This dataset provides a direct comparison between early (proliferation) and late (differentiation) stages of cochlear development.
Single-Cell and Developmental Expression Datasets	gEAR: Kelly [25] GEO: GSE137299	n = 11	This single-cell RNA-sequencing dataset encompasses a developmental timeline spanning embryonic day 14 (E14) through postnatal day 7 (P7) in wild-type mice, with a total of 11 samples distributed across these timepoints.
	gEAR: Hertzano [26] GEO: GSE64543	n = 18	This dataset comprises expression data from distinct cell populations isolated from newborn (P0) Atoh1-GFP transgenic mice. The study isolated three distinct cell types from both cochlear and vestibular tissues: hair cells, epithelial non-sensory cells (supporting cells), and non-epithelial cells. The dataset includes a total of 18 samples with comprehensive biological replication: three biological replicates for each of the three cell types, from both cochlear and vestibular tissues.
	gEAR: He [27] GEO: GSE111347	n = 12	This dataset provides detailed transcriptomic profiles of specific cell types isolated from the adult mouse cochlea, with a particular focus on supporting cells. RNA was extracted from four distinct cell populations: two types of supporting cells (Deiters’ cells and pillar cells) and two types of sensory cells (Inner Hair Cells and Outer Hair Cells). The cells were collected from adult CBA/J mice using the suction pipette technique, with 1000 cells collected for each biological replicate of pillar and Deiters’ cells. The experimental design included comprehensive replication: three biological replicates for Deiters’ cells and pillar cells (each with two technical repeats), two biological replicates for Inner Hair Cells, and three biological replicates for Outer Hair Cells (each with two technical repeats). All samples were sequenced on the Illumina HiSeq 2500 platform. The dataset includes a total of 12 samples, covering Deiters’ cells (samples 1–6) and pillar cells (samples 1–6).
	gEAR: Avraham [28] N/A	N/A	This dataset contains RNA-sequencing data from the sensory epithelia of the inner ear collected from C57Bl/6J mice at two critical developmental timepoints: embryonic day 16.5 (E16.5) and postnatal day 0 (P0) to examine both cochlear and vestibular tissues to capture developmental gene expression changes across these distinct inner ear compartments. Note that detailed information regarding the exact number of samples and specific experimental conditions is not provided in the available metadata.
	gEAR: Chen [29] GEO: GSE60019	n = 18	This dataset provides comprehensive transcriptomic profiles of hair cells and surrounding cells isolated from the developing mouse inner ear across multiple developmental stages. The experimental design spans four key developmental timepoints: embryonic day 16 (E16), postnatal day 0 (P0), postnatal day 4 (P4), and postnatal day 7 (P7). The dataset includes 18 samples in total, representing both positive (hair cells, designated with “p”) and negative (surrounding cells, designated with “n”) cell populations from cochlea (C) and utricle (U) at each developmental stage. Some conditions have additional biological replicates (e.g., P0Cp-1, P0Cp-2), enhancing the statistical robustness of the data.
	gEAR: Udea 2023 [30] GEO: GSE233487	n = 5	This dataset provides single-cell RNA-sequencing profiles of SOX2-expressing cells isolated from human inner ear organoids across a developmental time course. The experimental design captures five distinct timepoints spanning a critical window of inner ear organoid development: differentiation days 20, 30, 40, 50, and 60.

To validate whether these developmental expression patterns are conserved in human cochlear development, we examined their expression in human cochlear organoids from day 20 to day 60 of differentiation (Figure 5D). In these human organoids, the human genes *TP53* and *EZH2* showed progressive reduction in expression levels across various cell types. Notably, the human gene *ZBTB4* displayed a distinct pattern with peak expression in hair and supporting cells around day 50, contrasting with its minimal expression in progenitor populations.

Further quantitative analysis in mouse cochlear organoids, comparing day 7 (D7) and day 21 (D21), revealed significant changes in relative normalized expression (Figure 5E). The mouse genes *Trp53* and *Ezh2* showed significant downregulation from D7 to D21 (*p* < 0.05), with expression decreasing to 0.363-fold and 0.215-fold, respectively. Conversely, the mouse gene *Zbtb4* exhibited significant upregulation, increasing 1.941-fold by D21 (*p* < 0.05). These opposing expression dynamics in the mouse model suggest distinct regulatory roles during cochlear maturation, with *Trp53* and *Ezh2* potentially involved in early developmental processes, while *Zbtb4* may play a role in later maturation stages.

### 2.5. Temporal Dynamics of Key Signaling Pathways During Human Cochlear Organoid Differentiation

**Figure 6:**
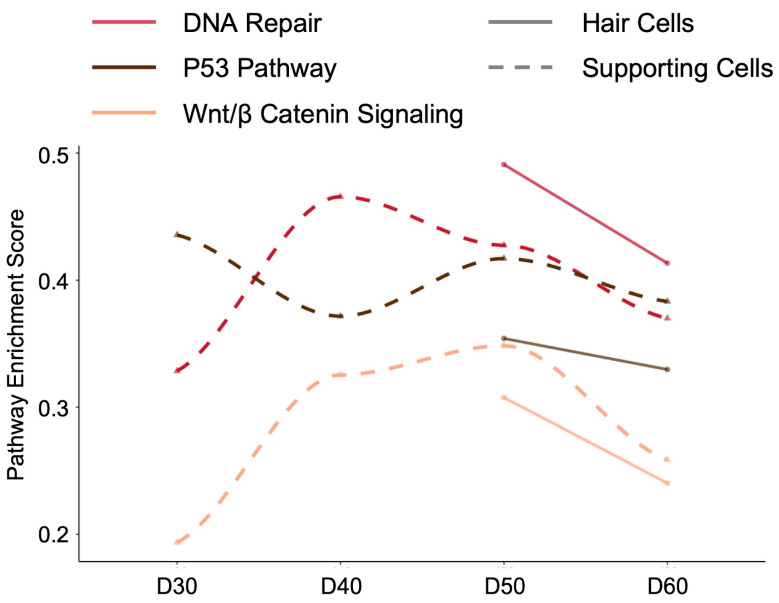
Temporal dynamics of key signaling pathways during human cochlear organoid differentiation. Pathway enrichment scores for DNA Repair (red), P53 Pathway (black), and Wnt/β-Catenin Signaling (orange) in hair cells (solid lines) and supporting cells (dashed lines) from day 30 to day 60 of differentiation from Udea 2023 dataset in gEAR portal. In supporting cells, DNA Repair pathway peaks at day 40, P53 pathway shows highest activity at day 30 with continuous decline, and Wnt/β-Catenin signaling increases from day 30 to peak at day 50. Hair cells demonstrate synchronized pathway activation with all three pathways reaching maximum enrichment at day 50, followed by sharp decline by day 60, suggesting a critical developmental checkpoint for terminal differentiation. These pathways were selected based on their direct functional relationships with the identified regulatory markers *Trp53*, *Ezh2*, and *Zbtb4*.

To understand the functional implications of our identified key human regulatory markers—*TP53*, *EZH2*, and *ZBTB4*—we examined the temporal dynamics of three closely related signaling pathways (DNA Repair, P53 Pathway, and Wnt/β-Catenin Signaling) during human cochlear organoid differentiation from day 30 to day 60 (Figure 6). For a comprehensive view of all hallmark pathway activities across different human cell types and developmental time points, see the pathway enrichment heatmap in Supplementary Figure S5.

In human supporting cells (dashed lines), each pathway exhibited distinct temporal patterns. The DNA Repair pathway peaked at day 40 (enrichment score ~0.47) before progressively declining through days 50 and 60. The P53 pathway, directly linked to human *TP53* function, showed highest activity at day 30 (enrichment score ~0.45) followed by continuous downregulation until day 60. In contrast, Wnt/β-Catenin signaling displayed an opposite pattern, starting at its lowest point at day 30 (enrichment score ~0.2), rising to peak at day 50 (~0.35), and then declining by day 60. These dynamics suggest that human supporting cells undergo a developmental transition from DNA damage response and P53-mediated regulation in early stages to Wnt signaling-driven maturation processes.

Human hair cells (solid lines) demonstrated a more synchronized pattern across all three pathways. DNA Repair, P53, and Wnt/β-Catenin pathways all reached their maximum enrichment at day 50 (enrichment scores ranging from ~0.35 to 0.5), followed by a sharp decline by day 60. This coordinated peak at day 50 suggests a critical developmental window where human hair cells engage multiple signaling mechanisms—likely involving the regulatory functions of *TP53* (P53 pathway), *EZH2* (chromatin remodeling affecting multiple pathways), and *ZBTB4* (transcriptional regulation)—before transitioning to a more mature state.

The contrasting temporal patterns between human supporting cells and hair cells reflect their different developmental trajectories. While supporting cells show sequential pathway activation and deactivation, hair cells exhibit synchronized pathway regulation, particularly at day 50. The dramatic decrease in all pathway activities by day 60 in hair cells coincides with their functional maturation.

## 3. Discussion

In this study, we extracted EVs at different developmental stages during the development of otic progenitor cells into organoids and compared EV miRNAs derived from the proliferation and differentiation-stage organoids. EVs were extracted at the time of proliferation when hair cells were not yet formed and at the time of differentiation when hair cells and sterocilia were visible and mechanical channels were observed. There were 35 miRNAs that changed significantly at both time points, of which 6 miRNAs were significantly upregulated in the differentiation stage, while 29 miRNAs showed significant downregulation, suggesting a predominant reduction in specific miRNA secretion during the transition from proliferation to differentiation.

When the differentially expressed miRNAs were categorized into groups involved in cochlear development, the heatmap analysis showed that each miRNA was involved in multiple pathways, and, among them, mmu-let-7b-3p, mmu-miR-455-3p, and mmu-miR-574-5p were more involved in various pathways than others. The let-7 miRNA family, highly conserved across bilaterians, regulates developmental timing [31,32,33,34]. Let-7 also acts as a tumor suppressor by directly targeting oncogenes such as RAS, HMGA2, and c-Myc [35,36,37]. In humans, 12 genomic loci encode let-7 family members: let-7a-1, let-7a-2, let-7a-3, let-7b, let-7c, let-7d, let-7e, let-7f-1, let-7f-2, let-7g, let-7i, and miR-98. In our previous study, let-7a-5p, let-7e-5p, let-7f-5p, and let-7g-5p were upregulated during the proliferation phase, whereas in the current experiment, let-7a-5p, let-7d-5p, let-7e-5p, and let-7f-5p were downregulated during the differentiation phase, with only let-7b-5p showing an increased expression-a pattern aligning with our earlier findings [21]. This divergent expression dynamics among let-7 isoforms (downregulation of let-7a/d/e/f-5p coupled with let-7b-5p upregulation) suggests a stage-specific regulatory shift, fine-tuning cellular processes during the transition from proliferative to differentiated states. The Lin28/let-7 axis forms a dual-negative feedback loop that regulates cell-cycle progression: Lin28 suppresses let-7 maturation, sustaining proliferative capacity via upregulation of Cyclin D1 and CDK4 while blocking differentiation signals [38,39,40]. Mechanistically, elevated let-7b directly targets the 3′UTR of Lin28, disrupting this feedback loop and driving auditory progenitor differentiation. This shift facilitates cell-cycle exit, enhances hair cell maturation, and reduces progenitor proliferation in the developing inner ear sensory epithelium.

The human protein–protein interaction network analysis identified TP53 as the central regulatory hub, interacting with other crucial human factors including EZH2 and ZBTB4. Notably, these three genes exhibited opposing temporal expression patterns that were conserved across species. In human organoids, the human genes *TP53* and *EZH2* were downregulated during development, while *ZBTB4* was upregulated. This same pattern was observed in our organoid models, where the mouse orthologs *Trp53* and *Ezh2* were downregulated, and *Zbtb4* was upregulated.

This divergent regulation indicates a sophisticated developmental program where early proliferative signals mediated by *TP53* (in humans) and *Trp53* (in mice), as well as *EZH2* (in humans) and *Ezh2* (in mice), must be attenuated. Concurrently, late differentiation factors like *ZBTB4* (in humans) and *Zbtb4* (in mice) must be activated to permit proper inner ear tissue maturation. The integration of our miRNA data with the protein interaction network suggests that EV miRNAs may function as key extracellular signaling molecules that coordinate the activity of these master regulators across different cell populations during cochlear development.

The conservation of expression patterns for the human genes *TP53*, *EZH2*, and *ZBTB4* and their respective mouse orthologs, *Trp53*, *Ezh2*, and *Zbtb4*, between mouse models and human cochlear organoids suggests evolutionary preservation of these regulatory mechanisms in inner ear development. Our pathway analysis human organoids (Figure 6) further revealed that these human genes orchestrate their functions through distinct but interconnected signaling cascades.

The temporal dynamics of DNA Repair, P53, and Wnt/β-Catenin pathways showed remarkable cell-type specificity: human supporting cells exhibited sequential pathway transitions (early P53 activity transitioning to late Wnt signaling), while human hair cells demonstrated synchronized pathway activation peaking at day 50. This coordinated regulation in hair cells, coinciding with peak human *ZBTB4* expression, suggests a critical developmental checkpoint where multiple signaling mechanisms converge to drive terminal differentiation. The dramatic decline in all pathway activities by day 60 marks the completion of this differentiation program, highlighting how the miRNA-mediated regulation of the *TP53*, *EZH2*, and *ZBTB4* gene network (and its mouse counterparts) controls the precise timing of cochlear maturation through modulation of their associated signaling networks.

Our conclusions regarding EV-miRNA-mediated regulation are correlative and derived from target prediction, pathway enrichment, and concordant expression patterns across cochlear datasets. We have not yet demonstrated that EV-delivered miRNAs are necessary and/or sufficient to drive the observed pathway changes in recipient otic cells or quantified the contribution of EVs relative to other extracellular miRNA pools. Accordingly, we have tempered causal language and explicitly note this limitation.

## 4. Materials and Methods

### 4.1. Cochlear Cell Isolation

All animal experiments were approved by the Institutional Animal Care and Use Committee of Korea University College of Medicine (Korea 2024-0042). Temporal bones were removed from postnatal day 2 mice (ICR), and cochleae were harvested. The bony cochlea, spiral ganglion, and stria vascularis were removed, and the organ of Corti was obtained. Then, we dissociated the organ of Corti into single cells and incubated them in 0.25% trypsin-EDTA (Thermo Fisher Scientific, Waltham, MA, USA) at 37 °C and 5% CO_2_ for 15 min. After the addition of a trypsin inhibitor (10 mg/mL) (Gibco) and DNase I (1 mg/mL) (Stemcell Technologies, Vancouver, BC, Canada), the tissue was mechanically dissociated and passed through a 70 μm strainer to remove aggregates.

### 4.2. Organoid Culture

Dissociated cells were seeded at densities of 2.5 × 10^4^ cells/mL in 12-well suspension culture dishes and maintained in proliferation medium at 37 °C and 5% CO_2_. Half of the medium was replaced every other day, and the cells were cultured for 7 days. The proliferation medium was composed of DMEM/F-12 (Thermo Fisher Scientific) containing N-2 supplement (Thermo Fisher Scientific), B-27 supplement (Thermo Fisher Scientific), and 100 μg/mL ampicillin. Small molecules and growth factors were also included in the proliferation medium: 20 ng/mL EGF (R&D systems, Minneapolis, MN, USA), 10 ng/mL FGF2 (R&D systems), 50 ng/mL IGF1 (R&D systems), 3 μM CHIR99021 (LC Laboratories, Woburn, MA, USA), 500 μM valproic acid (Sigma-Aldrich, St. Louis, MO, USA), 100 μg/mL 2-phospho-L-ascorbic acid (Sigma-Aldrich), and 2 μM TGF-β RI kinase inhibitor (Sigma-Aldrich) [20]. On day 7, the proliferation-stage organoids were transplanted into a 20%-Matrigel (R&D Systems) dome and cultured for seven more days under the same medium conditions, and then they were changed to a differentiation medium and cultured for another seven days. The differentiation medium was composed of DMEM/F-12 (Thermo Fisher Scientific) containing N-2 supplement (Thermo Fisher Scientific), B-27 supplement (Thermo Fisher Scientific), and 100 μg/mL ampicillin. Small molecules, 3 μM CHIR99021 (LC Laboratories) and 5 μM LY411575 (Sigma-Aldrich), were included in the differentiation medium (Figure 1).

### 4.3. Immunohistochemistry and Confocal Microscopy

On day 7 and 21, the organoids were immunohistologically evaluated with antibodies Myo7A, SOX2, and PAX2. Organoids were fixed with 4% paraformaldehyde for 30 min. The fixed specimens were permeabilized with 0.5% Triton X-100 in PBS for 30 min, blocked in a 5% BSA solution, and incubated overnight with antibodies specific for Myo7A (Proteus BioSciences, Waltham, MA, USA), SOX2 (Santa Cruz Biotechnology, Dallas, TX, USA), and PAX2 (Abcam, Cambridge, UK) at 4 °C. The organoids were washed three times for 15 min each with 0.2% Triton X-100 in PBS and incubated with the corresponding secondary antibodies at room temperature for 2 h. Stereocilia were stained using Phalloidin Labeling Probes (Thermo Fisher Scientific). Hair cells in organoids were labeled by brief exposure to FM1-43 FX (Invitrogen), a styryl dye used to study endocytosis and exocytosis. DAPI solution (Sigma-Aldrich) was subsequently added for 30 min to visualize cell nuclei. EdU incorporation during S-phase was revealed using an EdU assay (EdU-Click 647, Baseclick). The samples were imaged with an LSM800 confocal laser scanning microscope (Zeiss, Oberkochen, Germany), and images were processed with with ZEN 3.0 SR (Zeiss) software.

### 4.4. Organoid EV Isolation and Characterization

We collected 5th–7th and 19th–21st day culture media and isolated EVs using a commercially available kit (Exo2DTM-EV isolation kit, EXOSOMEplus, Seoul, Republic of Korea). Collected media were centrifuged at 3000× *g* for 15 min at 4 °C to remove cell debris. The supernatant was mixed with Exo2D^TM^ reagent and incubated at 4 °C for one hour and then centrifuged at 3000× *g* for 30 min at 4 °C. The aqueous phase of Exo2D traps exosomes. This phase appears as a white pellet. The remainder was discarded. The exosome sample was resuspended in 100 μL PBS and immediately stored at −80 °C.

Characterization was performed by using NTA (Malvern Panalytical, Malvern, UK) and immunoblotting with anti-CD9, CD 63, HSP70, and calnexin antibodies. The size and concentration of the exosomes were determined using a NanoSight™ LM10-HS10 system (Malvern Panalytical). A monochromatic laser beam (405 nm) and NanoSight™ tracking software version 3.0 was used to analyze the average exosome size and concentration.

A 40 μL exosome sample was mixed with 20 μL of RIPA buffer, heated with Laemmli buffer at 100 °C for 5 min, and subjected to sodium dodecyl sulfate polyacrylamide gel electrophoresis. The isolated proteins were transferred to a nitrocellulose membrane. The membrane was blocked with 5% skim milk and incubated overnight with antibodies for CD9 (Santa Cruz), anti-CD 63 (Santa Cruz), HSP70 (abcam), calnexin (Novus Biologicals, Centennial, CO, USA). After three washes with TBST, the membranes were incubated with peroxidase-conjugated secondary antibodies for two hours and washed again. The membranes were then treated with a chemiluminescent solution (Gendepot, Baker, TX, USA), and a ChemiDOC Touch Imaging System (Bio-Rad, Hercules, CA, USA) was used to detect the bound antibody signals.

### 4.5. Organoid EV-Derived Small RNA Sequencing

RNA extracted from EV was processed for sequencing library construction using the SMARTer smRNA-Seq Kit (Takara Bio, Kusatsu, Shiga, Japan) following the manufacturer’s protocol. Libraries were validated using an Agilent Bioanalyzer (Agilent Technologies, Palo Alto, CA, USA) and sequenced on an Illumina NovaSeq (Illumina, Inc., San Diego, CA, USA) platform. For bioinformatic analysis, raw reads were pre-processed by trimming the first 3 nucleotides and removing adapter sequences using cutadapt [41], retaining reads longer than 18 bp. Processed reads were clustered, filtered to remove ribosomal RNA sequences, and analyzed for known miRNAs using miRDeep2 [42] software with miRBase v22.1 [43] as reference. Novel miRNAs were predicted by mapping reads to the Mus musculus reference genome (mm10) using Bowtie 1.1.2 [44], followed by secondary structure prediction with the RNAfold algorithm integrated in miRDeep2.

### 4.6. Bioinformatics Analysis of EV miRNAs

Pathway analysis of differentially expressed EV miRNAs was performed using the miRPathDB [45] “Heat map calculator” tool with Gene Ontology—Biological Processes as reference. Target mRNAs were identified from the miRTarBase database [46], and protein–protein interaction networks were constructed using STRING-db [47] with a confidence score cutoff of 0.7. Networks were visualized in Cytoscape v3.10.3 [48], focusing on the largest subnetwork for detailed analysis. Functional enrichment analysis identified associated Gene Ontology biological processes, from which we extracted nodes specifically related to cell differentiation processes. The final EV miRNA-associated network was established by identifying the protein node with the highest connectivity and its adjacent interactions within the differentiation-related subnetwork.

### 4.7. Validation Using Public Transcriptome Datasets

We integrated two public RNA-sequencing datasets (GSE132635 [23] and GSE243581 [24], described in Table 1) by normalizing count matrices to TPM values and applying batch correction using the ComBat algorithm [49]. Pseudotime analysis was performed with monocle3 [50] to establish developmental trajectories, followed by dimensionality reduction through PCA and UMAP visualization. Proliferation stage samples served as root cells for pseudotime ordering, allowing classification into early differentiation (ED) and late differentiation (LD) groups. To examine cell-type-specific and developmental expression patterns of target genes, we utilized six publicly available datasets from the Gene Expression Analysis Resource (gEAR; https://umgear.org/NIHL, accessed on 20 February 2025) [51], including datasets from Kelly [25], Hertzano [26], He [27], Avraham [28], and Chen [29] for hearing studies and Ueda 2023 [30] for inner ear organoid differentiation. Expression profiles were accessed and visualized through the gEAR platform.

### 4.8. qPCR Analysis

Total RNA was extracted from day 7 and 21 organoids harvested at the end of culture using RNeasy plus mini kit (Qiagen, Hilden, Germany). The quality and concentration of the extracted RNA were assessed using NanoDrop 2000 (Thermo Fisher Scientific). Synthesis of complementary DNA was performed with 1 μg of total RNA using reagents in the cDNA Synthesis Kit (Bio-Rad).

qPCR was performed using the SsoAdvanced™ Universal SYBR^®^ Green Supermix (Bio-Rad) in the CFX Opus 96 Real-Time PCR System (Bio-Rad) according to the manufacturer’s instructions. Primers used for qPCR were as follows: mouse Trp53(transformation related protein 53), 5′-GAGTATCTGGAAGACAGGCAG-3′ and 5′-GATGATGGTAAGGATAGGTCGG-3′; mouse Ezh2 (enhancer of zeste 2 polycomb repressive complex 2), 5′-CCGAATAACAGTAGCAGACCC-3′ and 5′-ATTTGCTTCAGAGGAGCTGG-3′; Zbtb4 (zinc finger and BTB domain containing 4), 5′-CAGGTACCAGTGCATCTTCTG-3′ and 5′-TAGGAGGCCAGGGCTAATG-3, and mouse GAPDH, 5′-GTGCTGAGTATGTCGTGGAG-3′ and 5′-ATTTCTCGTGGTTCACACCC-3′. The expression level of the target genes was measured as a fold change and evaluated using the 2^−ΔΔCt^ method. Expression levels of the target genes were normalized to mouse GAPDH.

### 4.9. Single-Cell RNA-seq Pathway Analysis

Single-cell RNA-seq data from human cochlear organoids, “Ueda et al., 2023” [30], were analyzed using Seurat v4 with log-normalization, clustering, and UMAP visualization. Pathway activity scores for the Hallmark gene set collection (MSigDB [52]) were calculated as a mean expression of pathway genes per cell, analyzing only pathways with ≥3 detected genes. Pathway scores were compared across developmental time points (days 20–60) and cell types using Kruskal–Wallis tests with Benjamini–Hochberg correction. Hair cells and supporting cells were specifically analyzed for DNA Repair, P53 Pathway, and Wnt/β-Catenin Signaling pathways, with spatiotemporal dynamics visualized using line plots and loess smoothing.

### 4.10. Phase Assignment and Cross-Dataset Comparison

Phase labels were assigned using each dataset’s native staging system without re-quantifying canonical markers. Mouse data were staged by embryonic/postnatal day; organoid data by protocol day and cell-type annotations; RT-qPCR data by days-in-culture. Cross-dataset comparisons were based on developmental state equivalence rather than chronological synchronization, with no cross-species temporal normalization applied. Detailed time point-to-phase mappings are provided in Supplementary Table S2.

### 4.11. Statistical Analysis

Statistical comparisons between groups were performed using Student’s *t*-test. All statistical analyses were conducted using R version 4.0. All *p*-values presented in this study are two-sided, with statistical significance defined as *p* < 0.05 unless otherwise specified.

## 5. Conclusions

Our study demonstrated that EV-derived miRNAs are timely regulated during the proliferation and differentiation of inner ear organoids. There was a predominant reduction in specific miRNAs during the transition from proliferation to differentiation, and miRNA-related key regulatory genes are presumed to control the precise timing of inner ear maturation through modulation of their associated signaling networks. Our analysis provides insights into regulatory mechanisms that promote inner ear development, which could not only facilitate the optimization of organoid-based models for translational research and therapeutic development but be leveraged in miRNA-based therapeutic approaches.

## Data Availability

All data are publicly available. The NGS dataset in this study is available in the NCBI BioProject repository (accession: PRJNA1276453) available at https://www.ncbi.nlm.nih.gov/sra/PRJNA1276453 (accessed on 28 October 2025).

## Supplementary Materials

The supporting information can be downloaded at https://www.mdpi.com/article/10.3390/ijms262110627/s1. References [9,23,53,54,55,56,57] are cited in the supplementary materials.

## References

[1] Janesick A.S., Heller S. (2018). Stem Cells and the Bird Cochlea—Where Is Everybody?. Cold Spring Harb. Perspect. Med..

[2] Rubel E.W., Furrer S.A., Stone J.S. (2013). A brief history of hair cell regeneration research and speculations on the future. Hear. Res..

[3] Li W., Wu J., Yang J., Sun S., Chai R., Chen Z.-Y., Li H. (2014). Notch inhibition induces mitotically generated hair cells in mammalian cochleae via activating the Wnt pathway. Proc. Natl. Acad. Sci. USA.

[4] Oshima K., Grimm C.M., Corrales C.E., Senn P., Monedero R.M., Géléoc G.S.G., Edge A., Holt J.R., Heller S. (2006). Differential Distribution of Stem Cells in the Auditory and Vestibular Organs of the Inner Ear. J. Assoc. Res. Otolaryngol..

[5] White P.M., Doetzlhofer A., Lee Y.S., Groves A.K., Segil N. (2006). Mammalian cochlear supporting cells can divide and trans-differentiate into hair cells. Nature.

[6] Kubota M., Scheibinger M., Jan T.A., Heller S. (2021). Greater epithelial ridge cells are the principal organoid-forming progenitors of the mouse cochlea. Cell Rep..

[7] Lee S., Kubota M., Park E., Heller S., Im G.J., Chang J. (2025). Analysis of miRNAs from Inner Ear Organoid-Derived Extracellular Vesicles. J. Assoc. Res. Otolaryngol..

[8] Koehler K.R., Nie J., Longworth-Mills E., Liu X.-P., Lee J., Holt J.R., Hashino E. (2017). Generation of inner ear organoids containing functional hair cells from human pluripotent stem cells. Nat. Biotechnol..

[9] Jeong M., O’Reilly M., Kirkwood N.K., Al-Aama J., Lako M., Kros C.J., Armstrong L. (2018). Generating inner ear organoids containing putative cochlear hair cells from human pluripotent stem cells. Cell Death Dis..

[10] Koehler K.R., Mikosz A.M., Molosh A.I., Patel D., Hashino E. (2013). Generation of inner ear sensory epithelia from pluripotent stem cells in 3D culture. Nature.

[11] Van Niel G., D’Angelo G., Raposo G. (2018). Shedding light on the cell biology of extracellular vesicles. Nat. Rev. Mol. Cell Biol..

[12] Kowal J., Arras G., Colombo M., Jouve M., Morath J.P., Primdal-Bengtson B., Dingli F., Loew D., Tkach M., Théry C. (2016). Proteomic comparison defines novel markers to characterize heterogeneous populations of extracellular vesicle subtypes. Proc. Natl. Acad. Sci. USA.

[13] Melton C., Judson R.L., Blelloch R. (2010). Opposing microRNA families regulate self-renewal in mouse embryonic stem cells. Nature.

[14] Pauli A., Rinn J.L., Schier A.F. (2011). Non-coding RNAs as regulators of embryogenesis. Nat. Rev. Genet..

[15] Wang Y., Baskerville S., Shenoy A., E Babiarz J., Baehner L., Blelloch R. (2008). Embryonic stem cell–specific microRNAs regulate the G1-S transition and promote rapid proliferation. Nat. Genet..

[16] Chadly D.M., Best J., Ran C., Bruska M., Woźniak W., Kempisty B., Schwartz M., LaFleur B., Kerns B.J., Kessler J.A. (2018). Developmental profiling of microRNAs in the human embryonic inner ear. PLoS ONE.

[17] Patel M., Hu B.H. (2012). MicroRNAs in inner ear biology and pathogenesis. Hear. Res..

[18] Jiang P., Ma X., Han S., Ma L., Ai J., Wu L., Zhang Y., Xiao H., Tian M., Tao W.A. (2022). Characterization of the microRNA transcriptomes and proteomics of cochlear tissue-derived small extracellular vesicles from mice of different ages after birth. Cell. Mol. Life Sci..

[19] McLean W.J., Yin X., Lu L., Lenz D.R., McLean D., Langer R., Karp J.M., Edge A.S. (2017). Clonal Expansion of Lgr5-Positive Cells from Mammalian Cochlea and High-Purity Generation of Sensory Hair Cells. Cell Rep..

[20] Kubota M., Heller S. (2021). Murine cochlear cell sorting and cell-type-specific organoid culture. STAR Protoc..

[21] Choo O.-S., Park J.M., Park E., Chang J., Lee M.Y., Lee H.Y., Moon I.S., Lee K.-Y., Song J.-J., Nam E.-C. (2025). Consensus Statements on Tinnitus Assessment and Treatment Outcome Evaluation: A Delphi Study by the Korean Tinnitus Study Group. J. Korean Med. Sci..

[22] Lenz D.R., Gunewardene N., Abdul-Aziz D.E., Wang Q., Gibson T.M., Edge A.S.B. (2019). Applications of Lgr5-Positive Cochlear Progenitors (LCPs) to the Study of Hair Cell Differentiation. Front. Cell Dev. Biol..

[23] Kalra G., Lenz D., Abdul-Aziz D., Hanna C., Basu M., Herb B.R., Colantuoni C., Milon B., Saxena M., Shetty A.C. (2023). Cochlear organoids reveal transcriptional programs of postnatal hair cell differentiation from supporting cells. Cell Rep..

[24] Liu Q., Zhang L., Chen Z., He Y., Huang Y., Qiu C., Zhu C., Zhou D., Gan Z., Gao X. (2024). Metabolic Profiling of Cochlear Organoids Identifies α-Ketoglutarate and NAD(+) as Limiting Factors for Hair Cell Reprogramming. Adv. Sci..

[25] Kolla L., Kelly M.C., Mann Z.F., Anaya-Rocha A., Ellis K., Lemons A., Palermo A.T., So K.S., Mays J.C., Orvis J. (2020). Characterization of the development of the mouse cochlear epithelium at the single cell level. Nat. Commun..

[26] Elkon R., Milon B., Morrison L., Shah M., Vijayakumar S., Racherla M., Leitch C.C., Silipino L., Hadi S., Weiss-Gayet M. (2015). RFX transcription factors are essential for hearing in mice. Nat. Commun..

[27] Liu H., Chen L., Giffen K.P., Stringham S.T., Li Y., Judge P.D., Beisel K.W., He D.Z.Z. (2018). Cell-Specific Transcriptome Analysis Shows That Adult Pillar and Deiters’ Cells Express Genes Encoding Machinery for Specializations of Cochlear Hair Cells. Front. Mol. Neurosci..

[28] Rudnicki A., Isakov O., Ushakov K., Shivatzki S., Weiss I., Friedman L.M., Shomron N., Avraham K.B. (2014). Next-generation sequencing of small RNAs from inner ear sensory epithelium identifies microRNAs and defines regulatory pathways. BMC Genom..

[29] Scheffer D.I., Shen J., Corey D.P., Chen Z.-Y. (2015). Gene Expression by Mouse Inner Ear Hair Cells during Development. J. Neurosci..

[30] Ueda Y., Nakamura T., Nie J., Solivais A.J., Hoffman J.R., Daye B.J., Hashino E. (2023). Defining developmental trajectories of prosensory cells in human inner ear organoids at single-cell resolution. Development.

[31] Großhans H., Johnson T., Reinert K.L., Gerstein M., Slack F.J. (2005). The temporal patterning microRNA let-7 regulates several transcription factors at the larval to adult transition in *C. elegans*. Dev. Cell.

[32] Pasquinelli A.E., Reinhart B.J., Slack F., Martindale M.Q., Kuroda M.I., Maller B., Hayward D.C., Ball E.E., Degnan B., Müller P. (2000). Conservation of the sequence and temporal expression of let-7 heterochronic regulatory RNA. Nature.

[33] Reinhart B.J., Slack F.J., Basson M., Pasquinelli A.E., Bettinger J.C., Rougvie A.E., Horvitz H.R., Ruvkun G. (2000). The 21-nucleotide let-7 RNA regulates developmental timing in *Caenorhabditis elegans*. Nature.

[34] Evsen L., Li X., Zhang S., Razin S., Doetzlhofer A. (2020). *let-7* miRNAs inhibit CHD7 expression and control auditory-sensory progenitor cell behavior in the developing inner ear. Development.

[35] Johnson S.M., Grosshans H., Shingara J., Byrom M., Jarvis R., Cheng A., Labourier E., Reinert K.L., Brown D., Slack F.J. (2005). RAS Is Regulated by the let-7 MicroRNA Family. Cell.

[36] Lee Y.S., Dutta A. (2007). The tumor suppressor microRNA *let-7* represses the HMGA2 oncogene. Genes Dev..

[37] Sampson V.B., Rong N.H., Han J., Yang Q., Aris V., Soteropoulos P., Petrelli N.J., Dunn S.P., Krueger L.J. (2007). MicroRNA Let-7a Down-regulates MYC and Reverts MYC-Induced Growth in Burkitt Lymphoma Cells. Cancer Res..

[38] Thornton J.E., Gregory R.I. (2012). How does Lin28 let-7 control development and disease?. Trends Cell Biol..

[39] Heo I., Joo C., Cho J., Ha M., Han J., Kim V.N. (2008). Lin28 Mediates the Terminal Uridylation of let-7 Precursor MicroRNA. Mol. Cell.

[40] Balzeau J., Menezes M.R., Cao S., Hagan J.P. (2017). The LIN28/let-7 Pathway in Cancer. Front. Genet..

[41] Martin M. (2011). Cutadapt removes adapter sequences from high-throughput sequencing reads. EMBnet. J..

[42] Friedländer M.R., Mackowiak S.D., Li N., Chen W., Rajewsky N. (2012). miRDeep2 accurately identifies known and hundreds of novel microRNA genes in seven animal clades. Nucleic Acids Res..

[43] Kozomara A., Griffiths-Jones S. (2013). miRBase: Annotating high confidence microRNAs using deep sequencing data. Nucleic Acids Res..

[44] Langmead B., Trapnell C., Pop M., Salzberg S.L. (2009). Ultrafast and memory-efficient alignment of short DNA sequences to the human genome. Genome Biol..

[45] Kehl T., Kern F., Backes C., Fehlmann T., Stöckel D., Meese E., Lenhof H.-P., Keller A. (2019). miRPathDB 2.0: A novel release of the miRNA Pathway Dictionary Database. Nucleic Acids Res..

[46] Cui S., Yu S., Huang H.-Y., Lin Y.-C., Huang Y., Zhang B., Xiao J., Zuo H., Wang J., Li Z. (2024). miRTarBase 2025: Updates to the collection of experimentally validated microRNA–target interactions. Nucleic Acids Res..

[47] Szklarczyk D., Kirsch R., Koutrouli M., Nastou K., Mehryary F., Hachilif R., Gable A.L., Fang T., Doncheva N.T., Pyysalo S. (2022). The STRING database in 2023: Protein–protein association networks and functional enrichment analyses for any sequenced genome of interest. Nucleic Acids Res..

[48] Shannon P., Markiel A., Ozier O., Baliga N.S., Wang J.T., Ramage D., Amin N., Schwikowski B., Ideker T. (2003). Cytoscape: A software environment for integrated models of Biomolecular Interaction Networks. Genome Res..

[49] Leek J.T., Johnson W.E., Parker H.S., Jaffe A.E., Storey J.D. (2012). The sva package for removing batch effects and other unwanted variation in high-throughput experiments. Bioinformatics.

[50] Trapnell C., Cacchiarelli D., Grimsby J., Pokharel P., Li S., Morse M., Lennon N.J., Livak K.J., Mikkelsen T.S., Rinn J.L. (2014). The dynamics and regulators of cell fate decisions are revealed by pseudotemporal ordering of single cells. Nat. Biotechnol..

[51] Milon B., Shulman E.D., So K.S., Cederroth C.R., Lipford E.L., Sperber M., Sellon J.B., Sarlus H., Pregernig G., Shuster B. (2021). A cell-type-specific atlas of the inner ear transcriptional response to acoustic trauma. Cell Rep..

[52] Subramanian A., Tamayo P., Mootha V.K., Mukherjee S., Ebert B.L., Gillette M.A., Paulovich A., Pomeroy S.L., Golub T.R., Lander E.S. (2005). Gene set enrichment analysis: A knowledge-based approach for interpreting genome-wide expression profiles. Proc. Natl. Acad. Sci. USA.

[53] Masuda M., Pak K., Chavez E., Ryan A.F. (2012). TFE2 and GATA3 enhance induction of POU4F3 and myosin VIIa positive cells in nonsensory cochlear epithelium by ATOH1. Dev. Biol..

[54] Masuda M., Dulon D., Pak K., Mullen L.M., Li Y., Erkman L., Ryan A.F. (2011). Regulation of POU4F3 gene expression in hair cells by 5′ DNA in mice. Neuroscience.

[55] Liu Q., Zhang L., Zhu M.S., Wan G. (2021). High-throughput screening on cochlear organoids identifies VEGFR-MEK-TGFB1 signaling promoting hair cell reprogramming. Stem Cell Rep..

[56] Singh J., Randle M.R., Walters B.J., Cox B.C. (2024). The transcription factor Pou4f3 is essential for the survival of postnatal and adult mouse cochlear hair cells and normal hearing. Front. Cell Neurosci..

[57] Roccio M., Perny M., Ealy M., Widmer H.R., Heller S., Senn P. (2018). Molecular characterization and prospective isolation of human fetal cochlear hair cell progenitors. Nat. Commun..

