# Supplementary material for: Identification of microRNA-Related Target Genes for the Development of Otic Organoids
Source: Int J Mol Sci. 2025 Oct 31;26(21):10627. doi: 10.3390/ijms262110627 (PMC12608458; doi:10.3390/ijms262110627)
Supplement: Supplementary file 1 [file ijms-26-10627-s001.zip › Supplementary Tables.docx]

Supplementary Table S1. Orthologs of differentially expressed mouse microRNAs

| **Differentially Expressed Mouse miRNA** | **Hairpin Name** | **Human Ortholog** | **Information Source** |
| --- | --- | --- | --- |
| mmu-let-7b-3p | microRNA let7b | hsa-let-7b-3p | https://www.informatics.jax.org/marker/MGI:2676794 |
| mmu-miR-204-3p | microRNA 204 | hsa-miR-204-3p | https://www.informatics.jax.org/marker/MGI:2676879 |
| mmu-miR-335-5p | microRNA 335 | hsa-miR-335-5p | https://www.informatics.jax.org/marker/MGI:3619348 |
| mmu-miR-214-3p | microRNA 214 | hsa-miR-214-3p | https://www.informatics.jax.org/marker/MGI:2676890 |
| mmu-miR-455-3p | microRNA 455 | hsa-miR-455-3p | https://www.informatics.jax.org/marker/MGI:3629649 |
| mmu-miR-301a-3p | #N/A | #N/A | #N/A |
| mmu-miR-93-5p | microRNA 93 | hsa-miR-93-5p | https://www.informatics.jax.org/marker/MGI:3619439 |
| mmu-miR-181b-5p | microRNA 181b-2 | hsa-miR-181b-2-5p | https://www.informatics.jax.org/marker/MGI:3618736 |
| mmu-miR-199b-5p | microRNA 199b | hsa-miR-199b-5p | https://www.informatics.jax.org/marker/MGI:2676865 |
| mmu-miR-31-5p | microRNA 31 | hsa-miR-31-5p | https://www.informatics.jax.org/marker/MGI:3619330 |
| mmu-miR-574-5p | microRNA 574 | hsa-miR-574-5p | https://www.informatics.jax.org/marker/MGI:3718549 |
| mmu-miR-24-3p | microRNA 24-2 | hsa-miR-24-2-3p | https://www.informatics.jax.org/marker/MGI:3618755 |
| mmu-let-7a-5p | microRNA let7a-1 | hsa-let-7a-1-5p | https://www.informatics.jax.org/marker/MGI:2676793 |
| mmu-miR-98-5p | microRNA 98 | hsa-miR-98-5p | https://www.informatics.jax.org/marker/MGI:3619441 |
| mmu-let-7d-5p | microRNA let7d | hsa-let-7d-5p | https://www.informatics.jax.org/marker/MGI:2676796 |
| mmu-let-7e-5p | microRNA let7e | hsa-let-7e-5p | https://www.informatics.jax.org/marker/MGI:2676797 |
| mmu-let-7f-5p | microRNA let7f-2 | hsa-let-7f-2-5p | https://www.informatics.jax.org/marker/MGI:2676799 |
| mmu-miR-664a-3p | #N/A | #N/A | #N/A |
| mmu-miR-382-5p | microRNA 382 | hsa-miR-382-5p | https://www.informatics.jax.org/marker/MGI:3619392 |
| mmu-miR-25-3p | microRNA 25 | hsa-miR-25-3p | https://www.informatics.jax.org/marker/MGI:3619266 |
| mmu-miR-23b-3p | microRNA 23b | hsa-miR-23b-3p | https://www.informatics.jax.org/marker/MGI:2676898 |

#N/A, not available

Supplementary Table S2. Developmental Ranging (Staging) of Cochlear Hair Cells.

| **Species** | **Developmental Stage** | **Time Point** | **Key Markers & Rationale** | **Source** |
| --- | --- | --- | --- | --- |
| Mouse | Prosensory/Early Specification | Mouse Embryonic Day  E12–E12.5 | ATOH1, SOX2: Hair cell progenitors commit to their fate and begin to express the master regulator of hair cell development, ATOH1, within the SOX2-positive prosensory domain. | Masuda et al., 2012 [1] |
|  |  | Mouse Organoids Day 0  (in vitro differentiation) | Lgr5, Sox2, Hes1: Expanded Lgr5+ progenitor cells represent a prosensory-like state, poised for differentiation. | Kalra et al., 2023 [2] |
|  | Hair Cell (HC) Onset | Mouse Embryonic Day  E13–E14.5 | Pou4f3: Expression of the critical transcription factor Pou4f3 is initiated shortly after Atoh1, marking the nascent hair cells before the appearance of definitive structural markers. | Masuda et al., 2012 [1] |
|  | Early Maturation | Mouse Embryonic Day  E15–Postnatal Day P3 | Myo7a, Prestin: Expression of definitive hair cell markers like Myo7a begins (E15), followed by maturation markers like Prestin (P0), indicating the start of terminal differentiation. | Masuda et al., 2012 [1]; Masuda et al., 2011 [3] |
|  |  | Mouse Organoids Day 10–18  (in vitro differentiation) | Myo7a, Pou4f3: In organoids, the hair cell marker Myo7a is first observed (DIV14), and the proportion of cells co-expressing it with Pou4f3 increases, recapitulating the early maturation stage. | Liu et al., 202d [4] |
|  | Late Maturation | Mouse Postnatal Day P12 onward | Hearing Onset: Hearing onset occurs (P12-14) and mature hearing is established (4 weeks). The continued expression of Pou4f3 is essential for the survival and functional maintenance of hair cells. | Singh et al., 2024 [5] |
|  |  | Mouse Organoids Day 20–26  (in vitro differentiation) | Kinocilium loss, Ctbp2: The kinocilium, a feature of immature hair cells, is lost (DIV24), and nearly all hair cells express the synaptic ribbon marker Ctbp2, indicating functional maturation. | Liu et al., 2021 [4] |
| Human | Prosensory / Early Specification | Gestational Week W8–10 | SOX2, p27Kip1, LGR5, ATOH1: The prosensory domain is established as a postmitotic, SOX2-positive region expressing progenitor markers like LGR5 and the master regulator ATOH1. | Roccio et al.,2018 [6] |
|  |  | Human Organoids Day 20  (in vitro differentiation) | SOX2, PAX2: Organoids at this stage form otic vesicles expressing pro-sensory markers, recapitulating the early specification phase before definitive hair cell markers appear. | Jeong et al., 2018 [7] |
|  | Hair Cell (HC) Onset | Gestational Week W11 | Faint MYO7A: The first definitive hair cell marker, MYO7A, becomes faintly detectable in the basal turn of the cochlea. POU4F3 expression is inferred to begin around this time. | Roccio et al., 2018 [6] |
|  | Early Maturation | Gestational Week W12– W 14 | Robust MYO7A, BRN3C (POU4F3), ESPIN: Hair cells become clearly identifiable with robust marker expression. Differentiation follows a base-to-apex gradient, and hair bundles begin to form. | Roccio et al., 2018 [6] |
|  |  | Human Organoids Day 30–60  (in vitro differentiation) | ATOH1, MYO7A, CTBP2, PSD95: Hair cells are clearly observed in organoids, expressing synaptic markers (CTBP2, PSD95) and showing immature hair bundle structures. | Liu et al., 2021 [4] |
|  | Late Maturation | Gestational Week W14 onward | Tip links: Tip links, critical structures for mechanotransduction between stereocilia, begin to be observed, which is a key indicator of functional maturation. | Jeong et al., 2018 [7] |
|  |  | Human Organoids Day 90  (in vitro differentiation) | PRESTIN, PARVALBUMIN: The characteristic outer hair cell (OHC) marker PRESTIN is expressed, and more mature stereocilia bundles and synaptic structures are observed, indicating a mature hair cell phenotype. | Jeong et al., 2018 [7] |

**Supplementary Reference**

1. Masuda, M.; Pak, K.; Chavez, E.; Ryan, A. F., TFE2 and GATA3 enhance induction of POU4F3 and myosin VIIa positive cells in nonsensory cochlear epithelium by ATOH1. *Dev Biol* **2012,** 372, (1), 68-80.

2. Kalra, G.; Lenz, D.; Abdul-Aziz, D.; Hanna, C.; Basu, M.; Herb, B. R.; Colantuoni, C.; Milon, B.; Saxena, M.; Shetty, A. C.; Hertzano, R.; Shivdasani, R. A.; Ament, S. A.; Edge, A. S. B., Cochlear organoids reveal transcriptional programs of postnatal hair cell differentiation from supporting cells. *Cell Rep* **2023,** 42, (11), 113421.

3. Masuda, M.; Dulon, D.; Pak, K.; Mullen, L. M.; Li, Y.; Erkman, L.; Ryan, A. F., Regulation of POU4F3 gene expression in hair cells by 5' DNA in mice. *Neuroscience* **2011,** 197, 48-64.

4. Liu, Q.; Zhang, L.; Zhu, M. S.; Wan, G., High-throughput screening on cochlear organoids identifies VEGFR-MEK-TGFB1 signaling promoting hair cell reprogramming. *Stem Cell Reports* **2021,** 16, (9), 2257-2273.

5. Singh, J.; Randle, M. R.; Walters, B. J.; Cox, B. C., The transcription factor Pou4f3 is essential for the survival of postnatal and adult mouse cochlear hair cells and normal hearing. *Front Cell Neurosci* **2024,** 18, 1369282.

6. Roccio, M.; Perny, M.; Ealy, M.; Widmer, H. R.; Heller, S.; Senn, P., Molecular characterization and prospective isolation of human fetal cochlear hair cell progenitors. *Nat Commun* **2018,** 9, (1), 4027.

7. Jeong, M.; O'Reilly, M.; Kirkwood, N. K.; Al-Aama, J.; Lako, M.; Kros, C. J.; Armstrong, L., Generating inner ear organoids containing putative cochlear hair cells from human pluripotent stem cells. *Cell Death Dis* **2018,** 9, (9), 922.
