# Supplementary material for: Identification of microRNA-Related Target Genes for the Development of Otic Organoids
Source: Int J Mol Sci. 2025 Oct 31;26(21):10627. doi: 10.3390/ijms262110627 (PMC12608458; doi:10.3390/ijms262110627)
Supplement: Supplementary file 1 [file ijms-26-10627-s001.zip › Supplementary_Material_Figure.pdf]

Identification of miRNA-related Target Genes for the Development of Otic Organoids

Sehee Lee, Sungjin Park *et al.*

Supplementary Material

Contents

Supplementary Figure S1. Heatmap of differentially expressed extracellular vesicle miRNAs between proliferation and differentiation stages.

Supplementary Figure S2. Gene ontology biological process enrichment analysis of differentially expressed EV miRNAs.

Supplementary Figure S3. Trajectory analysis of mouse inner ear organoid gene expression datasets for sample selection.

Supplementary Figure S4. Comprehensive protein-protein interaction network analysis of EV miRNA target genes.

Supplementary Figure S5. Comprehensive hallmark pathway enrichment analysis across cell types and developmental stages in human cochlear organoids.

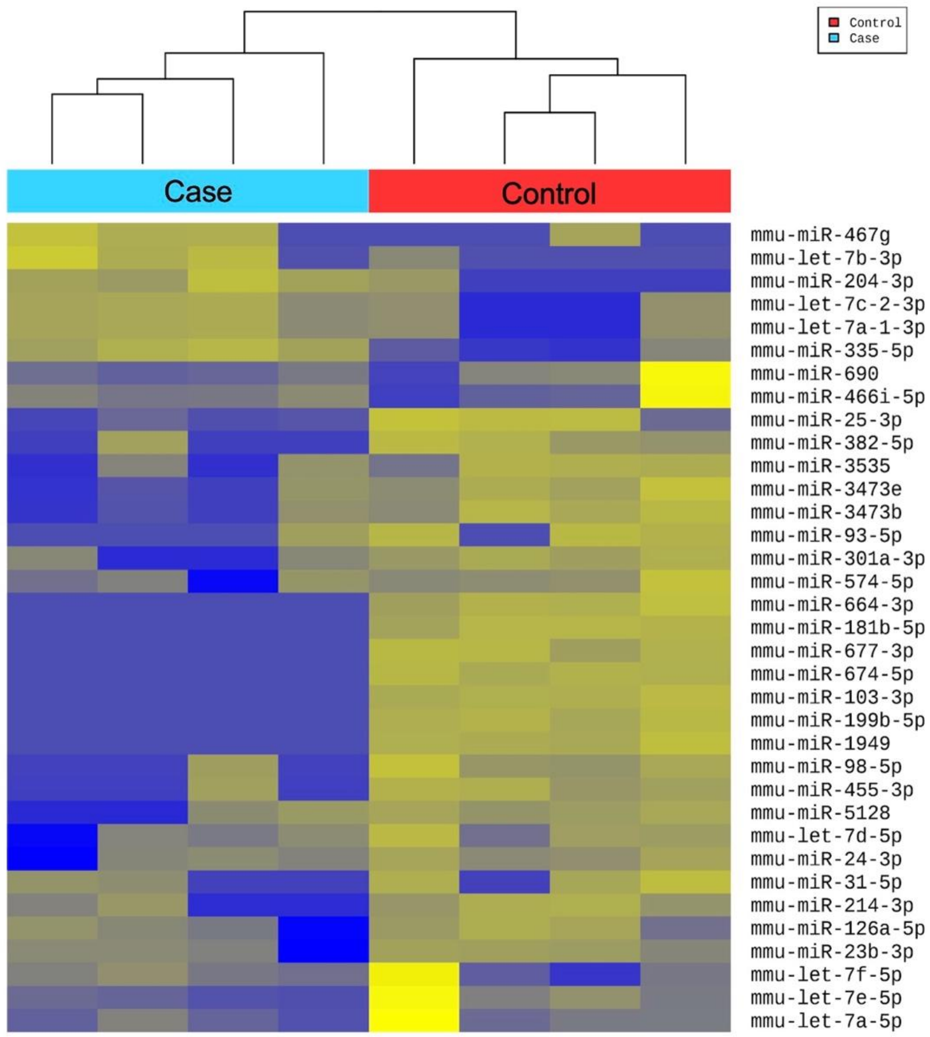

Supplementary Figure S1. Heatmap of differentially expressed extracellular vesicle miRNAs between proliferation and differentiation stages. Hierarchical clustering analysis showing expression patterns of 35 differentially expressed miRNAs in extracellular vesicles from control (proliferation stage) and case (differentiation stage) inner ear organoids. Blue indicates lower expression and yellow indicates higher expression. The dendrogram shows the clustering relationships between samples and miRNAs.

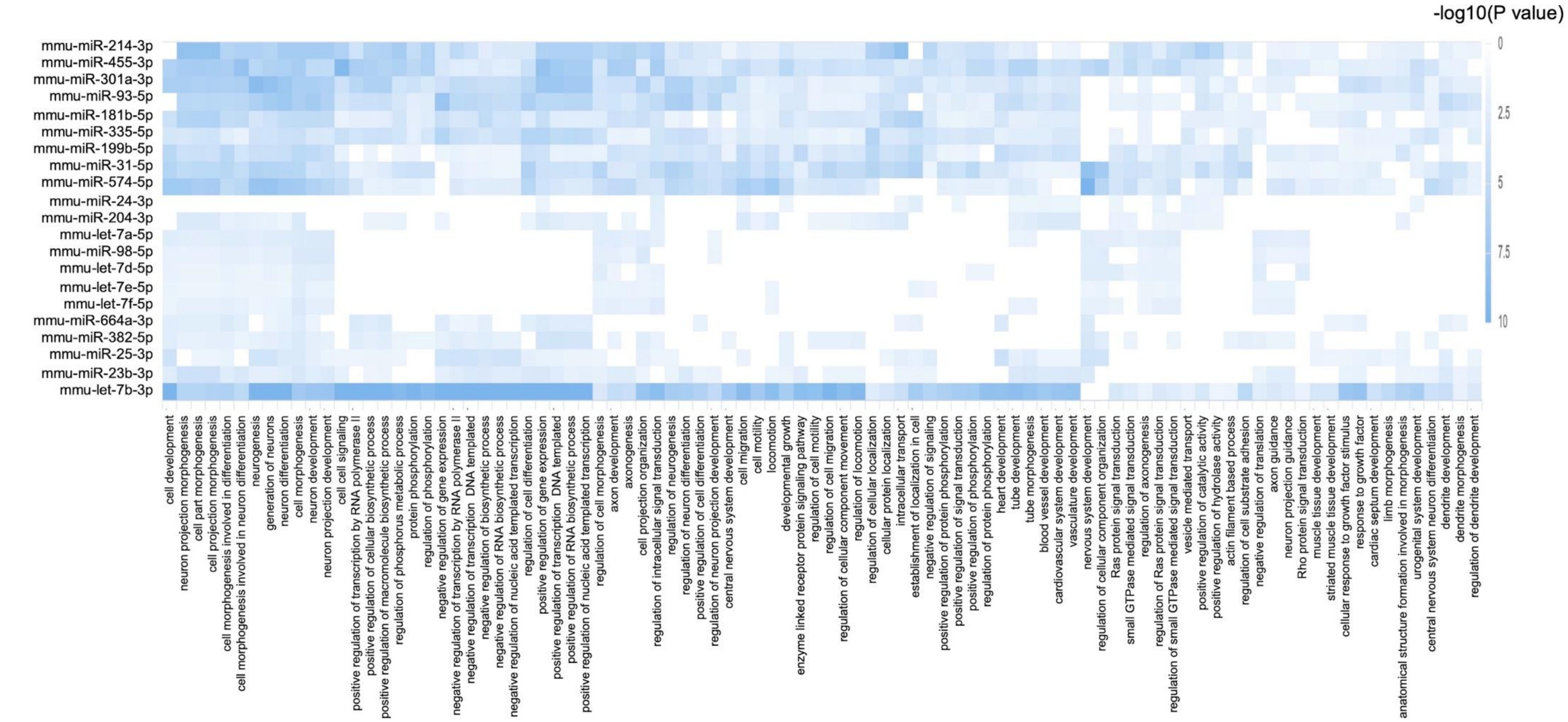

Supplementary Figure S2. Gene ontology biological process enrichment analysis of differentially expressed EV miRNAs. Heatmap displaying the association between 21 differentially expressed EV miRNAs (y-axis) and 92 significantly enriched gene ontology biological processes (x-axis). Color intensity represents -log10(P value), with darker blue indicating higher statistical significance. Biological processes are organized into functional categories including cell development, morphogenesis, differentiation, axon guidance, neurogenesis, and various signaling pathways.

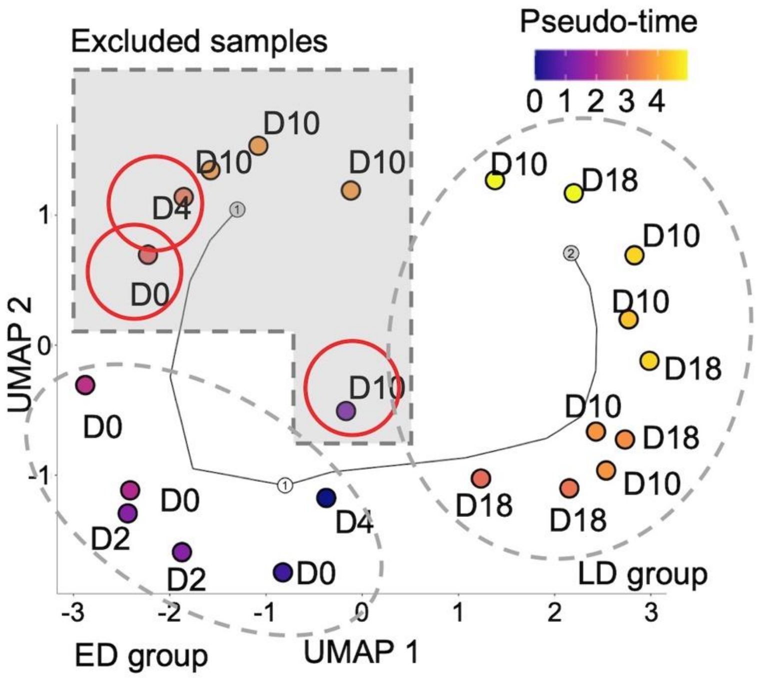

Supplementary Figure S3. Trajectory analysis of mouse inner ear organoid gene expression datasets for sample selection. UMAP visualization showing pseudo-time trajectory analysis of two mouse organoid gene expression datasets. Samples are colored by pseudo-time progression (purple to yellow) and labeled by differentiation day (D0-D18). The trajectory path connects samples in developmental order, with early differentiation (ED) group samples (D0-D4) and late differentiation (LD) group samples (D10-D18) indicated. Gray shaded areas indicate excluded samples not connected in the pseudo-time trajectory. Only samples along the connected trajectory were included in the bulk gene expression analysis shown in Figure 4E.

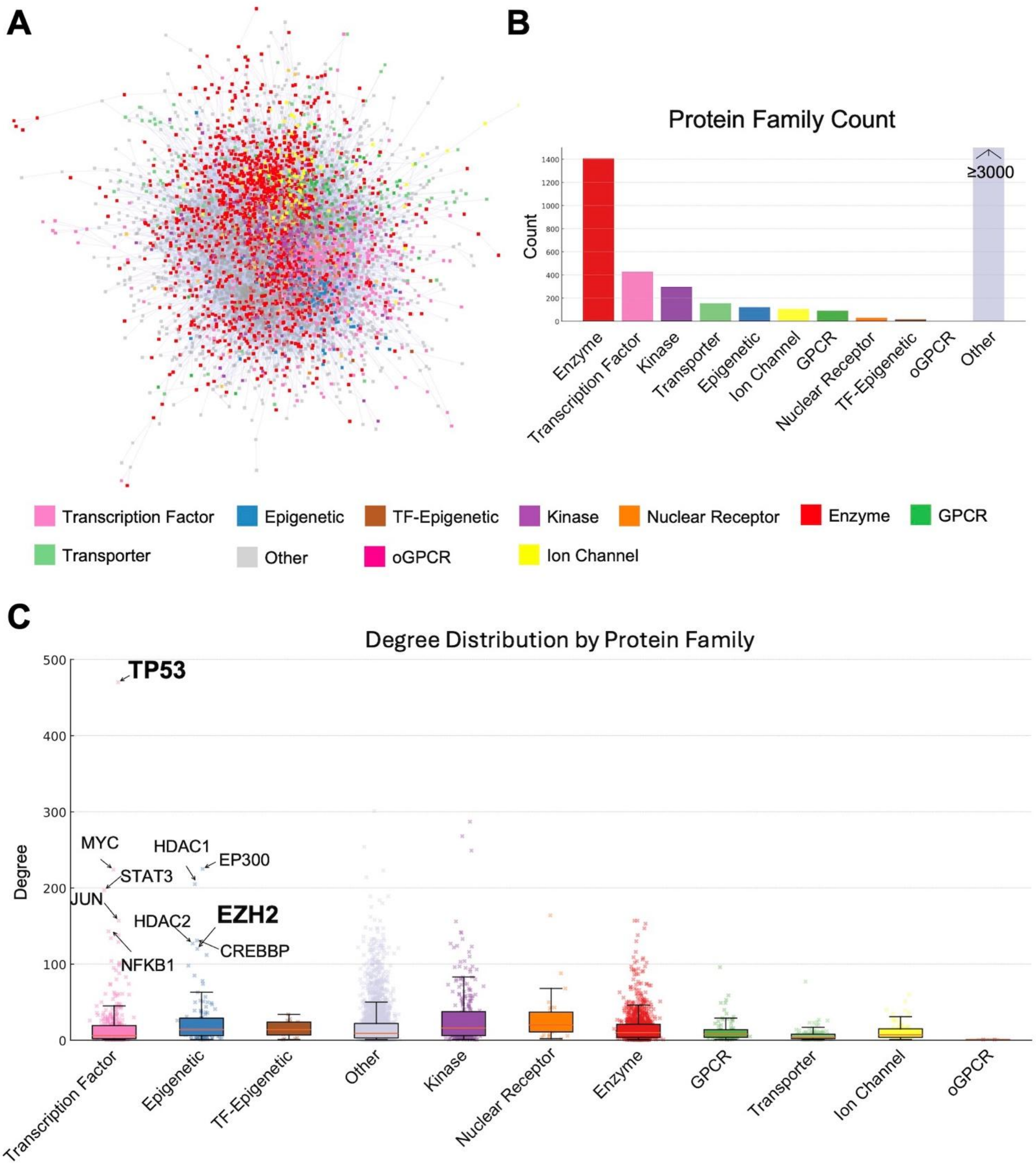

Supplementary Figure S4. Comprehensive protein-protein interaction network analysis of EV miRNA target genes. (A) Visualization of the largest submodule containing 5,900 protein nodes from the complete network of 8,468 target mRNAs regulated by differentially expressed EV miRNAs. Node colors represent different protein families. **(B)** Distribution of proteins by functional family within the network. Enzymes constitute the largest category (>1,400 proteins), followed by transcription factors, kinases, and transporters. The "Other" category comprises approximately

3,000 proteins with diverse functions. **(C)** Degree distribution analysis across different protein families showing network connectivity. TP53 emerges as the most highly connected hub with a degree of approximately 480, followed by MYC, HDAC1, STAT3, EP300, JUN, HDAC2, EZH2, CREBBP, and NFKB1. Box plots show the distribution of connectivity degrees for each protein family.

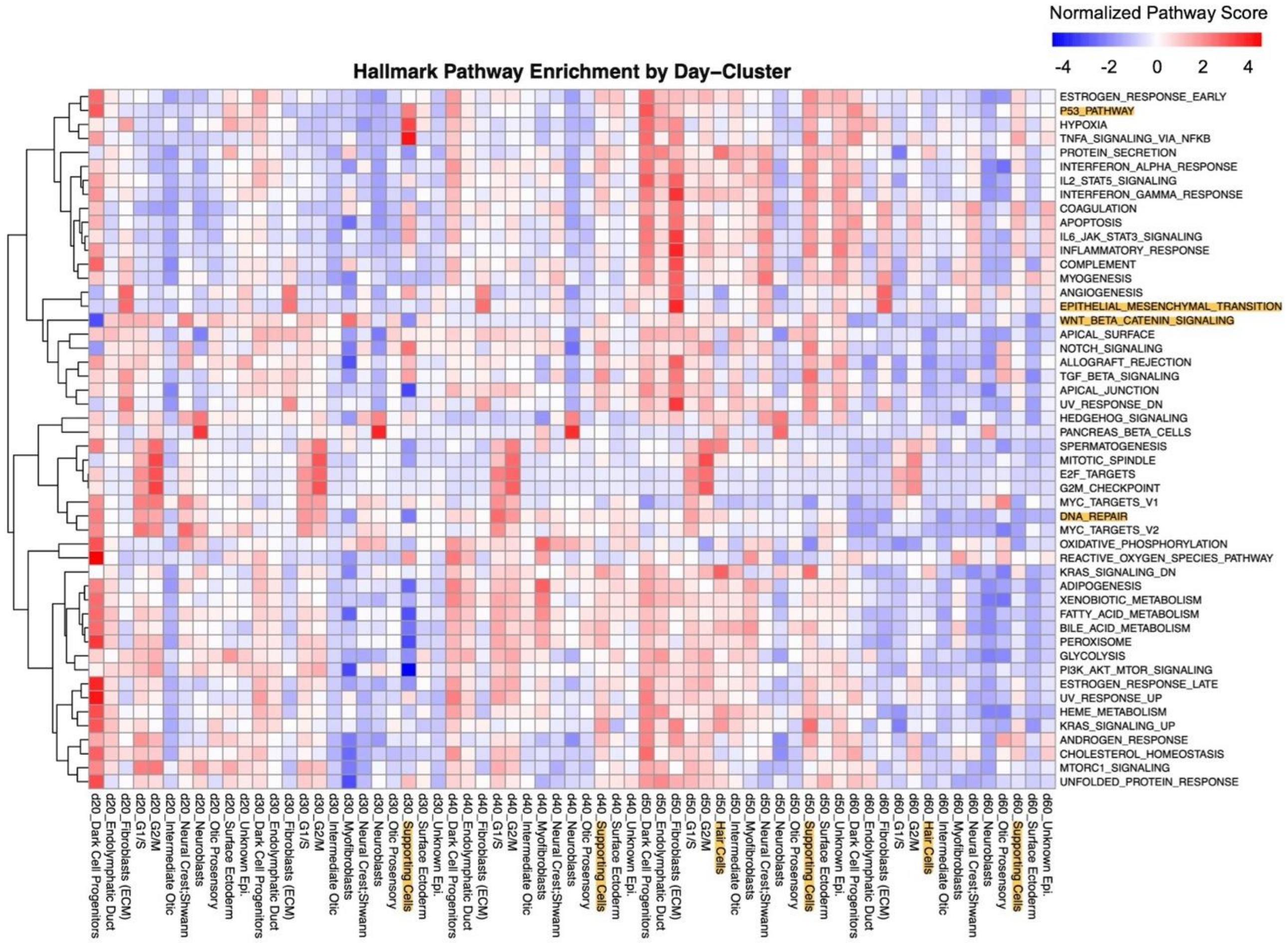

Supplementary Figure S5. Comprehensive hallmark pathway enrichment analysis across cell types and developmental stages in human cochlear organoids. Heatmap showing normalized pathway scores for 50 hallmark gene sets across different cell types from day 20 to day 60 of differentiation. Rows represent hallmark pathways hierarchically clustered by similarity, and columns represent cell type-day combinations. Red indicates pathway upregulation and blue indicates downregulation (normalized pathway score scale: -4 to 4). Key pathways highlighted in the main text (DNA Repair, P53 Pathway, and WNT_BETA_CATENIN_SIGNALING) are indicated. Cell types include dark cell progenitors, endolymphatic duct, fibroblasts, cell cycle phases (G1/S, G2/M), intermediate otic cells, myofibroblasts, neural crest/Schwann cells, neuroblasts, otic prosensory cells, supporting cells, hair cells, surface ectoderm, and unknown epithelial cells.
